# Subarachnoid Haemorrhage

**Published:** 1961-04

**Authors:** H. Lovell Hoffman

**Affiliations:** Neurologist, Bath Group of Hospitals


### SUBARACHNOID HAEMORRHAGE:

### Early assessment in a General Hospital

BY

H. LOVELL HOFFMAN, M.D., F.R.C.P.

*Neurologist, Bath Group of Hospitals*

The recent paper by McKissock and his colleagues is one of the most valuable contributions to the study of the treatment of sub-arachnoid haemorrhage that has yet appeared. (McKissock, Richardson and Walsh 1960). As a result of a carefully controlled trial, they are able to state that for ruptured aneurysms of the internal carotid artery at or near the origin of the posterior communicating vessel, carotid ligation in the neck produces results far superior to conservative treatment, provided that the patients are not unconscious or dying of an intra-cranial haematoma. The trial is still proceeding and it is anticipated that further pronouncements for aneurysms at other sites will be made later. These are awaited with great interest.

It may seem presumptuous to add a further contribution to the literature on sub-arachnoid haemorrhage at the present time. Any such communication that discussed the relative merits of medical and surgical treatment would most certainly deserve this criticism. It is generally agreed that the problem of the physician in a General Hospital, and of the general practitioner at the bedside, is to decide which patient should be investigated by angiography. In many areas this involves transfer to a neurosurgical unit where the requisite facilities exist. Brinton (1960) has reviewed the distribution of neurologists and neuro-surgeons throughout the country. A glance at his map, which indicates the situation of the various neuro-surgical units, will show that responsibility for making the decision is often considerable. In this paper, I shall discuss the early assessment of the patient, as did Holmes (1957 and 1958) and Henson (1957). A small unselected group of 154 cases admitted to two general hospitals is reported, which is considered to give a reasonably clear overall impression of subarachnoid haemorrhage as it presents in the practice of general medicine.

### MATERIAL

All case records were examined which were filed under the primary heading of subarachnoid haemorrhage in the two General Hospitals in Bath, namely the Royal United and St. Martin's Hospitals. The period covered was from 1st January, 1952, to the end of June, 1960. Some 70 cases were discarded; these were of traumatic haemorrhage, primary intra-cerebral haemorrhage, sub-arachnoid haemorrhage in infants, and those wrongly classified or in which the diagnosis was in doubt. Some cases with hypertension and hemiplegia which were included and appear in the "recovered" group were possibly due to primary intra-cerebral haemorrhage. It was felt, however, that their inclusion would cause the lesser error for the following reasons. It is known that dense hemiplegia can occur from ruptured aneurysms, especially those on the middle cerebral artery; hypertension predisposes to aneurysm formation, several hypertensive patients in the series being found at post mortem to have aneurysms (the ages of seven of these were over seventy); and finally, massive primary intra-cerebral haemorrhage has a generally accepted high mortality.

The investigation started as a purely domestic one with the object of comparing our mortality rate with that in other groups of cases. Certain points of wider interest have arisen and the series is accordingly worth publishing. Subjects were classified according to age, sex, type of treatment, death or survival, type of lesion, and presence or absence of hypertension or arterio-sclerosis. Only deaths that occurred in hospital are recorded in the tables and thus the usual follow-up period of about six weeks is unduly short. Some other authors have, however, adopted a similar follow-up period so that for the immediate mortality it is possible to make some comparison with their figures. Patients were classified as "hypertensive" when the blood pressure was above 155/100, or if there were clinical or post-mortem signs of gross arterio-sclerosis. An attempt was made to assess the blood pressure in a quiescent period if possible, sometimes from previous or subsequent out-patient notes.

### ANALYSIS OF MATERIAL

### *Sex Incidence*

There was a considerable preponderance of females over males. In the females the overall mortality was greater (see Table I).

### *The Effect of Hypertension*

In the conservatively treated cases, excluding those with angiomata, the mortality was 29 per cent for normotensive, and 43·5 per cent for hypertensive patients (see Table II).

TABLE I (All Cases)

| | | *Under* 55 | *Over* 55 | *Deaths* | *Mortality per cent* |
|---|---|---|---|---|---|
| Male | 57 | 26 | 31 | 17 | 29·8 |
| Female | 97 | 45 | 52 | 38 | 39 |
| | 154 | 71 | 83 | 55 | 35·7 |

TABLE II

Effect of Hypertension—(Conservatively treated cases excluding angiomata).

| | *Number of cases* | *Recovered* | *Died* | *Mortality per cent* |
|---|---|---|---|---|
| Hypertensive | 85 | 48 | 37 | 43·5 |
| Non-hypertensive | 48 | 34 | 14 | 29 |

TABLE III

(Numbers in parenthesis represent angiomata, and except where stated, these are excluded from the main figures.)

| | *Number of cases* | | *Deaths* | *Mortality per cent* |
|---|---|---|---|---|
| Conservative treatment | Under 55 (5) | 53 | 22 | 41·5 |
| | Over 55 (2) | 80 | 29 | 36·25 |
| | Total (7) | 133 | 51 | 38·3 |
| Surgical treatment | (1) | 13 | 4 | 30·7 |
| Total, Angiomata excluded | (8) | 146 | 55 | 37·7 |
| Total, Angiomata included | | 154 | 55 | 35·7 |

*Age, Type of Treatment, and Mortality*

The results are collected together in Table III. In this Table eight cases of angioma are separated from the others, as the prognosis and indications for treatment of these lesions deserve separate consideration (see below). The first striking fact revealed by the Table is the very low proportion of cases which were eventually treated by operation, namely less than 10 per cent of the whole series, and 18·3 per cent of the under-55 age group. As these patients were treated during a period when the author believed that all those under the age of 55, without hypertension, should be considered as candidates for operation, these figures were somewhat surprising. It was apparent that our handling of these patients might warrant criticism and that perhaps we had not adequately used the neurosurgical facilities available. This question will be discussed later.

Another unexpected finding is the lower immediate mortality of 36·25 per cent in the older age group as compared with that of 41·5 per cent in the younger one. No explanation for this can be offered. A higher mortality would be expected in the older age group and in fact is nearly always found by others. The overall immediate mortality is 36·4 per cent for conservatively treated cases when angiomata are included, and this is somewhat lower than 45–50 per cent which is the figure accepted by many authors. One reason for this is the method of selection of the present series by which rarer and fatal causes of subarachnoid haemorrhage are excluded. Such causes are, for example, blood diseases, subacute bacterial endocarditis, haemorrhage from malignant tumours, and primary intra-cerebral haemorrhage. There are, however, some indications that modern methods of conservative treatment may be reducing the mortality. Holmes (1958) gives an immediate mortality of 43 per cent for 100 cases so treated from 1936–1951. But for forty-two cases similarly treated from 1952–1957 he had only fourteen deaths, a mortality of 33.3 per cent. Although his two sets of cases are not strictly comparable, the last figure is, in fact, lower than that of the present series, treated over a similar period, namely from 1952 up to the present time.

### FURTHER CLINICAL ANALYSIS

*Surgically Treated Cases*

Fourteen patients were treated by operation, all in the regional neurosurgical unit at Frenchay Hospital, Bristol. There were four deaths. Operation was performed on only one patient over 55, a woman who had a successful right occipital lobectomy for an angioma and who was well 2½ years later with no disability except a left homonymous hemianopia. During the period under review the author and the neurosurgeons concerned agreed that usually patients over 55 should not be referred for operation. Henson (1957) was of the same opinion. For this reason the age of 55 is taken as an arbitrary division between the two age groups which appear in Table III and elsewhere. No further analysis of this group of cases or the results of operation will be made. The numbers are far too small to lead to any useful conclusions and the subject is outside the scope of this paper.

*Angiomata*

Seven cases of angioma and one of choroid plexus papilloma, which bled spontaneously, are separated in Table III because the prognosis and indications for treatment differ from those in cases of aneurysm. They are classified as "angiomata" and will be considered briefly.

Four patients had large inoperable angiomata, found by angiography, and are still alive. Two have hemiplegia and fits; one of these, a woman of 54, is severely disabled, but has looked after herself for 30 years, in spite of repeated fits and several haemorrhages. The other, a man of 27, had his first bleed twelve years after the lesion was discovered as a result of a hemiplegia and epilepsy; he has returned to work.

Two patients, a man of 45 and a woman of 57, who probably had their first haemorrhage 34 and 30 years ago respectively, are not seriously disabled.

These cases illustrate the comparatively good prognosis as regards life for some large arterio-venous angiomata. The bleeding is less likely to be catastrophic than that from aneurysms.

Two patients were discharged from hospital alive, but died later. One, a woman of 38, was found at autopsy to have an angioma at the junction of the anterior spinal with the left vertebral artery, which had not been revealed by a vertebral angiogram. The other, a girl of 20, was found by angiography to have a choroid plexus papilloma. She was discharged after her initial bleed but was later admitted direct to the neurosurgical unit where she died after an occipital lobectomy. Of the remaining two patients, one had the syndrome of spinal angioma which was confirmed by myelography. The other had an occipital angioma successfully removed by lobectomy.

*Treatment of Patients under 55*

It is proposed to analyse the treatment of these patients in some detail, because in this group it was felt that operation should be considered whenever possible. Under these circumstances the fact that only thirteen of seventy-one patients were so treated requires some explanation. Bath is fortunately situated, being only 12 miles from the neurosurgical unit in Bristol, and the ambulance journey is achieved in thirty minutes. The decision whether or not to transfer patients is, therefore, considerably easier than in more remote parts of the region where the distances involved may be up to 180 miles. In some cases, although angiography was carried out, operation was not performed either because the films were negative or because the lesion found was unsuitable for surgical treatment. But as every case submitted for angiography was thus given the chance of operation the cases will be analysed from this aspect.

In order to discover if full advantage had been taken of the available neurosurgical and radiological facilities, our experience of the fifty-eight conservatively-treated patients under the age of 55 may be analysed (this figure includes five patients with angioma).

(*a*) Angiograms were taken in twenty-four of these patients, including all those with angioma; and positive findings were recorded in twelve patients.

Eight of the patients *with positive angiograms* recovered; three had inoperable angiomata, one had a choroid plexus papilloma, and four had inoperable aneurysms.

Nine of the twelve patients *with negative angiograms* recovered, though one died later of a left vertebral artery angioma. Three died, all with aneurysms that had not been revealed by angiography.

(*b*) No angiogram was taken in thirty-four patients. Of these, nineteen recovered, twelve of whom were hypertensive. Of the fifteen patients who died, ten died within 48 hours of admission (or of the onset of haemorrhage), four were hypertensive, and one died after prolonged coma. There were two patients in this group whose treatment might have been different if angiography had been performed; one of them died shortly after leaving hospital, and the other was a woman of 54 who had already had two meningeal haemorrhages. Angiography was to have been carried out after a period of rest, but she died from a third bleed seven weeks later. There is no doubt that the decision to delay angiography in this case was an error of judgement, at a time when we were in two minds about the efficacy of surgical treatment in patients over 50.

*Treatment of patients over 55*

The eighty-two conservatively treated cases in the over-55 age group were similarly analysed. In seven of these, angiograms were taken but in those over 70 this investigation was not considered. Of the twenty-nine who died there was one non-hypertensive patient who, after a previous bleed at the age of 57, should perhaps have had an angiogram. Of the others, hypertension, early death, or the clinical state was considered to have precluded angiography or operation. Of the fifty-three who recovered there were twelve between 55 and 70 in whom age was the only reason for withholding angiography. Eight of these have been followed up for periods of 1 to 8 years and none died of a recurrent haemorrhage. Eight patients over 70 recovered: one of these with hypertension was alive 4 years later and, at the age of 77 was admitted to a geriatric ward for loneliness; a strange indictment of the success of our treatment.

### DISCUSSION

In this series there are only three cases of patients who died without angiography where, in retrospect, it is considered that the investigation should have been performed.

Thirteen of seventeen normotensive individuals, who were discharged without angiography, have been followed up for periods ranging from 1 to 8 years. Perhaps we were fortunate that all except one, aged 54, survived. Eight of these were in the 55 to 70 age group and the investigation was omitted on the ground of their age alone.

One reason for advising conservative treatment in patients over 55, a decision with which Henson (1957) agrees, was the fact that they tend to stand intracranial operations less well than younger persons. Another was the fact that cross-circulation is less likely to be adequate if carotid ligation should be necessary. This could have been proved or disproved by carrying out the Matas test of digital compression of each carotid artery in turn for 10 minutes. It was not, in fact, performed on patients under our care. It is of interest to note that in thirty-seven cases of posterior communicating aneurysm in which McKissock, Richardson and Walsh (1960) used carotid ligation, ten developed hemiplegia, and of these, eight were over the age of 50. No doubt, some additional indications for or against operation in the older age group will be forthcoming when McKissock and his colleagues publish their further observations. Until then, the author will tend to treat patients over the age of 55 conservatively, except where intra-cerebral clot is suspected, or in those who have had two or more bleeds. In the latter there is always the chance that an operable angioma may be present, as was indeed the case in the single patient over 55 who was submitted to operation.

If, as these figures suggest, angiography will be required in more than 50 per cent of cases under 55, the problem facing the physician without neuro-radiological facilities in his own hospital is to decide when to transfer these patients to a neuro-surgical unit. It is now generally agreed that operation should not be carried out in a comatose or stuporose patient, except in the presence of intracerebral clot. This should be suspected if there are signs of involvement of one cerebral hemisphere, for example hemiparesis, hemianopia, or dysphasia. If, in such a patient, stupor persists or becomes deeper, angiography should be performed as soon as possible. If the clot is revealed, its surgical removal may be life-saving (Small 1957). It should be remembered that therapeutic lumbar puncture or the removal of more than 1 or 2 ml. of cerebro-spinal fluid in these cases is dangerous, the space-occupying clot being liable to produce herniation of temporal lobes or cerebellum (Meadows 1951). Vascular spasm accompanying the haemorrhage is an even commoner cause of impaired consciousness and hemisphere involvement. It plays an extremely important part in subarachnoid bleeding (Small 1957), and is one of the most potent causes of death or permanent disability. The presence of clot or spasm can only be distinguished by arteriography, but frequently both are present in the same patient. Spasm, with its signs of stupor and hemisphere involvement, will tend to pass off during the two weeks after the haemorrhage, but while the operative risk becomes less, so the risk of recurrent haemorrhage increases, to reach its maximum during the second week when the protective spasm ceases to be effective.

The closest co-operation between the physician and the neurosurgeon is essential during this period. Patients who have had more than one haemorrhage are in great danger, and arteriography should be carried out as soon as possible. In the case of "first offenders" the following procedure has been adopted by the author. As soon as a patient has been selected as a possible case for operation, the neurosurgical unit is informed and requested to admit the patient within 7 to 10 days. The usual conservative treatment for subarachnoid haemorrhage is then carried out. This consists of absolute rest with one or two pillows, the patient being allowed to do nothing for himself. Feeding should be by nasal stomach tube if the state of consciousness makes this necessary. Regular turning is instituted and prophylactic antibiotics are given to prevent chest infection. If the latter is becoming a real danger, gentle percussion of the chest is permissible. The air-way must be kept clear by the use of an electric sucker, and tracheotomy may sometimes be required; it is always better done too early than too late. Restlessness is nearly always due to pain from the sterile meningitis induced by the effused blood, and it must be controlled. Pethilorphan is the drug of choice and may be given every three hours if necessary. If this is not effective and there are no signs of intracerebral clot, therapeutic lumbar puncture can be undertaken. If the C.S.F. pressure is over 250–300 mm, it may be slowly reduced by 100–150 mm. Even in the absence of signs of hemisphere involvement, clot may be present, and a relatively high protein content of the initial C.S.F. specimen should suggest caution. Sometimes hypothermia will be needed in very severe cases. It was used in one case, not under the author's care, where a man aged 60 became unconscious $2\frac{1}{2}$ hours after a diagnostic lumbar puncture. He regained consciousness after 24 hours treatment and was well nearly 2 years later. Another patient (not in the series) developed decerebrate spasms several days after his bleed. These were controlled by chlorpromazine and pethedine, 50 mgms of each, without cooling. This simplified his transfer to the neurosurgical unit where he recovered after ventricular drainage.

During the week of conservative treatment before the angiography which is advocated (Small 1957, Falconer 1958), a few patients will die of recurrent haemorrhage. This is a risk which must be accepted and three such deaths occurred in the under-55 age group. If the patients had been referred earlier, these deaths would undoubtedly have occurred in the neurosurgical unit, or perhaps actually during transfer, which might have been blamed for the occurrence. In point of fact, the writer cannot recall a case in which transfer to another hospital has been responsible for a fatal bleed. On the other hand, patients often improve considerably during the week when spasm is passing off, so that at the end of the period a previously poor surgical risk may have become a reasonably good one. An initially high blood pressure may fall during this period. Hypothalamic derangement (due to the haemorrhage) or perhaps raised intracranial pressure in some instances, are causes of temporary hypertension. It is therefore important to assess the hypertension with the aid of previous blood pressure readings if these are available, and to take into account the state of the retinal arteries, the findings in the urine, the presence or absence of cardiac failure, the size of the heart and perhaps the results of electrocardiography.

### CONCLUSIONS

The correct treatment of subarachnoid haemorrhage depends to a great extent on the selection of patients for operation. Our series suggests that more than 50 per cent of patients under 55 should be investigated by arteriography, but that only a proportion of these will finally be treated surgically. In the future, it may be that fewer rather than more cases of aneurysm so discovered will undergo operation. No final pronouncement has yet been made for aneurysms on the middle cerebral, anterior cerebral and anterior communicating arteries, or for those in the posterior fossa. In centres where arteriography is carried out almost exclusively in the neurosurgical unit, the wastage of neurosurgical beds utilized for patients treated without operation is appreciable. Unfortunately, it seems unlikely that an adequate number of neurosurgical units will be established for some considerable time. One is therefore forced to the conclusion that angiography and other neuroradiological investigations should be carried out in certain strategically situated general hospitals. These techniques would also be used in the investigation of cases of epilepsy of late onset, or suspected cerebral tumour. The procedures and the interpretation of results require considerable skill and experience, but these could be acquired by general radiologists with resultant economy in the occupation of neurosurgical beds. The neurologist, covering a wide area, would act as a liason between general physician, radiologist and neurosurgeon, and would be an essential member of the team for management of cases of spontaneous subarachnoid bleeding.

### SUMMARY

(1) An unselected group of 154 patients with subarachnoid haemorrhage has been analysed. These were admitted to two General Hospitals in a provincial city, and their early assessment and treatment has been discussed.

(2) It is considered that angiography will be necessary in more than 50 per cent of patients under the age of 55, but that only a proportion of these will eventually be treated by operation.

(3) The distance of many centres from a neurosurgical unit is stressed.

(4) It is therefore suggested that cerebral angiography should be carried out for these patients in selected General Hospitals, a close liason having been established between the general physician, neurologist, radiologist and neurosurgeon.

The author has great pleasure in thanking the Consultant Physicians of Bath for allowing him to use their cases in this study; Messrs. G. L. Alexander, D. G. Phillips, and A. Hulme for their ready help and cooperation during the past 12 years; and Dr. Gordon Thomson of the South-Western Neuro-surgical Unit who performed the majority of the angiograms.

### REFERENCES

Brinton, D. (1960). *Proc. roy. Soc. Med.*, **53**, 261.

Falconer, M. A. (1958). *Brit. med. J.*, **1**, 790.

Henson, R. A. (1957). *Proc. roy. Soc. Med.*, **50**, 284.

Holmes, J. McD. (1957). *Ibid.*, **50**, 281.

(1958). *Brit. med. J.*, **1**, 788.

McKissock, W., Richardson, A., and Walsh, L. (1960). *Lancet*, **1**, 1203.

Meadows, S. P. (1951). In: *Modern Trends in Neurology*, 1st series. Ed. A. Feiling. London: Butterworth.

Small, J. M. (1957). *Proc. roy. Soc. Med.*, **50**, 289.

